# Whole-Genome Re-Sequencing Identifies Male-Associated Markers and a Marker-Enriched Region on Chromosome 14 in *Siniperca scherzeri*

**DOI:** 10.3390/biology15191675

**Published:** 2026-09-22

**Authors:** Jianbo Zheng, Xiaohai Zhang, Xiaohui Xu, Shili Liu, Wenping Jiang, Chao Zhu, Cancan Huang, Fei Li

**Affiliations:** 1 Key Laboratory of Genetics and Breeding, Zhejiang Institute of Freshwater Fisheries, Huzhou 313001, China; 2 Yangtze Delta Region Institute of Tsinghua University, Zhejiang, Jiaxing 314006, China; 3 School of Fishery, Zhejiang Ocean University, Zhoushan 316022, China

**Keywords:** sexual dimorphism, molecular sex identification, chromosome 14, male-associated genomic interval, sex-control breeding

## Abstract

Sex-specific markers are important for genetic sex identification and sex-control breeding in aquaculture. *Siniperca scherzeri* (Steindachner, 1892) is an economically important freshwater fish with female-biased sexual size dimorphism, but its sex-associated genomic architecture remains poorly characterized. Here, whole-genome resequencing of 10 females and 10 males generated approximately 480 Gb of clean data and identified 55 candidate male-associated fragments using a read-presence/absence screening strategy. PCR testing identified seven candidate male-associated markers that showed male-specific amplification and complete concordance with phenotypic sex in the tested *S. scherzeri* cohort. Marker4, Marker5, and Marker18 showed the same amplification pattern in the tested *Siniperca chuatsi* (Basilewsky, 1855) and *Siniperca knerii* (Garman, 1912) samples. The seven amplicons mapped to a marker-enriched interval at 20.677–20.695 Mb on chromosome 14. Marker18 overlapped a partial candidate *amh*-family gene model; however, no male-specific sequence variant, sex-biased expression, or functional role was tested. Because all *S. scherzeri* samples originated from one farmed cohort of unknown pedigree and the PCR assays lacked dedicated no-template and internal amplification controls, the observed concordance should not be interpreted as established diagnostic performance. The data support a male-associated marker cluster on chromosome 14, rather than establishing a Y-specific sex-determining region or a differentiated sex chromosome. Independent population validation with appropriate assay controls, together with genome-wide structural, expression, and functional analyses, is required.

## Simple Summary

Spotted mandarin fish is an important farmed freshwater species in East Asia, and females grow considerably larger than males. Producing all-female populations could therefore improve aquaculture efficiency. However, males and females are morphologically similar, making early sex identification difficult. We compared whole-genome sequence data from male and female spotted mandarin fish and identified seven candidate male-associated markers that showed male-specific amplification in the tested cohort. Three markers showed the same amplification pattern in the tested samples of two related species, *Siniperca chuatsi* and *Siniperca knerii*. The homologous reference positions of all seven markers clustered within a small interval on chromosome 14, and Marker18 overlapped a partial *amh*-family gene model. These observations nominate this interval for further investigation but do not establish a sex chromosome, a sex-determining region, or a causal sex-determining gene. The markers should be regarded as research-stage candidates because they were evaluated in one farmed cohort of unknown pedigree and the original PCR assays lacked dedicated no-template and internal amplification controls. Independent validation with appropriate controls is required before breeding application.

## 1. Introduction

Sexual dimorphism is widespread among cultured fishes and may be manifested in body size, coloration, growth rate, physiological traits (e.g., metabolic or endocrine characteristics), and behavioral traits (e.g., feeding or courtship behavior) [1,2,3]. When one sex exhibits economically advantageous traits, sex-control breeding can potentially improve production efficiency, stock uniformity, and economic returns in aquaculture [4,5,6]. However, unlike birds and mammals, teleost fishes exhibit highly diverse and evolutionarily labile sex-determination systems, and their sex chromosomes are often poorly differentiated and difficult to distinguish using conventional morphological or cytogenetic approaches [7,8,9]. Reliable sex-specific DNA markers are therefore valuable for early genetic sex identification and the development of mono-sex breeding programs, particularly in species whose phenotypic sex cannot be determined before sexual maturation [10,11,12].

A variety of molecular approaches have been used to develop sex-specific markers in aquaculture species, including amplified fragment length polymorphism, simple sequence repeats, randomly amplified polymorphic DNA, transcriptome sequencing, restriction-site-associated DNA sequencing, and whole-genome resequencing [13,14,15,16,17]. Compared with traditional marker-discovery methods, next-generation sequencing enables more efficient genome-wide detection of sex-associated SNPs, insertion/deletion variants, structural variants, and presence/absence sequences [18,19,20]. Whole-genome resequencing has therefore become an effective approach for developing sex-associated markers and generating hypotheses about the genomic basis of sex determination in non-model fishes [4,21]. Nevertheless, read-presence/absence screening is primarily a marker-discovery approach and, when used alone, cannot delimit a sex-determining region or establish the identity of a causal sex-determining gene.

*Siniperca scherzeri* (Steindachner, 1892), commonly known as the spotted mandarin fish, is an economically important freshwater carnivorous species of the family Sinipercidae [22]. It is native to East Asia, occurring in freshwater systems in China, the Korean Peninsula, and northern Vietnam [23,24]). Both wild capture and commercial aquaculture have been reported for this species [25]. Its global conservation status was assessed as Data Deficient (DD) in the IUCN Red List, indicating that available information was insufficient to determine its extinction risk [26]. Owing to its desirable flesh quality, high market value, and disease resistance, *S. scherzeri* has attracted increasing attention as a promising freshwater aquaculture species in China [27]. Although it grows more slowly than *S. chuatsi* under comparable rearing conditions, *S. scherzeri* has been reported to possess higher muscle-fibre density and collagen content [28], and its market price may be substantially higher than that of *S. chuatsi* [29]. In addition, females exhibit a growth advantage over males, making this species a potential candidate for the development of all-female production systems [30]. Because males and females are morphologically similar before sexual maturation, reliable molecular markers are required for early genetic sex identification.

Previous genomic studies have already provided important information about sex-associated regions in mandarin fishes. Ref. [31] used a pool-sequencing strategy to characterize male-biased genomic regions and develop male-specific markers in several mandarin fish species. More recently, ref. [30] performed whole-genome resequencing of 25 male and 25 female *S. scherzeri* individuals and combined genome-wide association analysis with *F_ST_* analysis. That study proposed chromosome 14 as the sex-linked chromosome, identified a 0.44-Mb putative sex-linked region at 19.34–19.78 Mb, and developed one male-specific and one female-specific marker. Consequently, the association of chromosome 14 with sex and the availability of sex-specific markers have already been reported in this species.

Nevertheless, additional independently identified markers may provide complementary loci for genetic sex identification because reliance on a single marker can be affected by population polymorphism, allele dropout, or PCR failure. A panel containing several male-associated markers may potentially provide greater assay redundancy, although this potential advantage requires evaluation in independent populations. In addition, the cross-species applicability of newly identified markers and the distribution of male-associated presence/absence sequences relative to the previously proposed sex-linked region remain incompletely characterized. Accordingly, the present study used separate whole-genome sequencing libraries from 10 male and 10 female *S. scherzeri* individuals and a read-presence/absence screening strategy to identify additional male-associated sequences. Candidate markers were assessed through two-stage PCR testing, followed by preliminary cross-species testing in *S. chuatsi* and *S. knerii*. The specific objectives were to develop a complementary panel of male-associated markers, evaluate their preliminary cross-species applicability, and determine their homologous positions in the available reference genome. The study was not designed to re-identify chromosome 14, redefine the previously proposed sex-determining region, or identify a master sex-determining gene.

## 2. Materials and Methods

### 2.1. Experimental Samples and Ethics Statement

A total of 90 sexually mature *S. scherzeri* individuals, including 45 males and 45 females, were used in this study. These farmed, two-year-old broodstock were purchased from a family farm in Quzhou, Zhejiang Province, China, in 2025. Their mean body weight was 420 ± 16.2 g. Sex was confirmed by gonadal examination. All individuals were obtained from the same farmed cohort; however, pedigree and family-relationship records were unavailable. Of these individuals, 10 males and 10 females were used for whole-genome resequencing, 5 males and 5 females for first-round PCR screening, 20 males and 20 females for expanded PCR evaluation, and 8 males and 8 females as the *S. scherzeri* reference group for preliminary cross-species testing. The remaining 2 males and 2 females were retained as backup samples and were not included in the molecular analyses. These sample groups were mutually exclusive, with no overlap among the analyses. In addition, 16 *S. chuatsi* (8 males and 8 females) and 16 *S. knerii* (8 males and 8 females) were sampled from the same farm in 2025 for preliminary cross-species testing. Their sex was also confirmed by gonadal examination. Sample codes and their corresponding analyses were provided in Supplementary Table S1. All animal-related experimental procedures in this study were carried out following established scientific guidelines and received approval from the Animal Ethics Committee of the Zhejiang Institute of Freshwater Fisheries to guarantee ethical compliance and animal welfare (Approval No. ZJIFF20210305).

### 2.2. DNA Extraction and Quality Assessment

Genomic DNA was extracted from muscle tissues using a commercial genomic DNA extraction kit (CoWin Biosciences, Taizhou, China) according to the manufacturer’s instructions. The quality and concentration of genomic DNA were examined by 1.0% agarose gel electrophoresis and NanoDrop spectrophotometer (Thermo Fisher Scientific, Waltham, MA, USA). DNA samples meeting the quality criteria of OD260/280 = 1.8–2.0 and OD260/230 ≥ 2.0 were selected for subsequent whole-genome resequencing and PCR testing.

### 2.3. Library Construction and Whole Genome Re-Sequencing

Qualified genomic DNA from each of the 10 female and 10 male individuals was used to construct a separate sequencing library. Thus, a total of 20 individual libraries were prepared, and no male or female DNA pooling was performed. Libraries were prepared using the TruSeq Nano DNA Library Prep Kit (Illumina, San Diego, CA, USA) according to the manufacturer’s instructions. Briefly, approximately 6 μg of genomic DNA from each individual was fragmented by ultrasonication, followed by end repair, A-tailing, adapter ligation, PCR enrichment, and size selection using AMPure XP beads (Beckman Coulter, Brea, CA, USA). The libraries were quantified using a Qubit 4 Fluorometer (Thermo Fisher Scientific, Waltham, MA, USA) and sequenced on an Illumina NovaSeq X Plus platform (Illumina, San Diego, CA, USA) using 150-bp paired-end reads. SsF1 and SsM1 were two of the 20 individually sequenced samples and were sequenced at greater depth for the construction of separate female and male de novo assemblies. The other 18 libraries comprised nine females and nine males and were used in the subsequent read-presence/absence screening. The theoretical sequencing coverage of each library was estimated by dividing the number of clean bases by the haploid reference-genome size of *S. scherzeri* (757,906,621 bp; GCA_055815275.1; [32]).

### 2.4. Screening of Candidate Male-Associated Fragments

Raw reads from all 20 individual libraries were quality-filtered using fastp (version 0.23.4)to remove adapter sequences, bases below Q20, reads containing ≥10% unknown bases, and reads shorter than 25 bp after trimming. The high-depth reads from SsM1 and SsF1 were assembled separately using SPAdes (version 3.15.5) [33], producing a male-derived de novo assembly and a female-derived de novo assembly, respectively. SsM1 and SsF1 were reserved for these assembly steps. Clean reads from the remaining 18 independently sequenced individuals, comprising nine males and nine females, were then mapped to the SsM1 male-derived assembly using BWA-MEM (version 0.7.17-r1188) with default parameters. Sequences covered by reads from all nine mapping-stage males but not covered by reads from any of the nine mapping-stage females were retained as candidate male-associated sequences. Candidate sequences that aligned to the SsF1 female-derived assembly were subsequently removed. This workflow yielded 55 candidate male-associated fragments for PCR screening. The analysis was designed to identify read-presence/absence sequences and did not include genome-wide SNP-based differentiation, structural-variant detection, or linkage-disequilibrium analysis.

### 2.5. PCR Evaluation of Candidate Male-Associated Fragments

To evaluate the 55 candidate male-associated sequences, primer pairs were designed using Primer Premier 6.0 and synthesized by Sangon Biotech Co., Ltd. (Shanghai, China) (Table S2). A two-step PCR screening strategy was used. All primer pairs were first tested in five phenotypic males and five phenotypic females. Primer pairs showing clear male-specific amplification were then tested in an independent expanded set of 20 males and 20 females. PCR was conducted in a 20 μL reaction containing 10 μL of 2× Es-Taq buffer, 1 μL of each primer (2.5 μM), 1 μL of genomic DNA (50 ng/μL), and sterile water. Cycling conditions were 95 °C for 5 min; 32 cycles of 95 °C for 1 min, 56 °C for 30 s, and 72 °C for 1 min; followed by 72 °C for 7 min. Products were examined by 1.5% agarose gel electrophoresis. The original PCR assays did not include a dedicated no-template control or a co-amplified internal amplification control. For the expanded cohort, descriptive within-cohort amplification sensitivity, amplification specificity, and overall concordance were calculated from the 2 × 2 sex-by-amplification table. Association with phenotypic sex was assessed using a two-sided Fisher exact test. Because all seven markers showed complete separation with zero discordant cells, the uncorrected odds ratio was infinite and is reported as complete separation rather than as a finite estimate. These statistics describe only the tested cohort and were not treated as estimates of population-level diagnostic performance.

## 3. Results

### 3.1. Statistics of Whole-Genome Resequencing Data

In this study, whole-genome resequencing was performed on 10 female and 10 male *S. scherzeri* individuals to identify candidate male-associated sequences. In total, 1,605,781,348 raw reads were generated, and 1,593,378,764 clean reads, corresponding to approximately 480 Gb of clean bases, were retained after quality filtering. The Q20 and Q30 values ranged from 98.71% to 98.99% and from 93.95% to 95.14%, respectively, while the GC content ranged from 40.81% to 41.49% (Table 1). Based on the 757,906,621-bp haploid reference genome assembly of *S. scherzeri* (GCA_055815275.1), the estimated sequencing coverage ranged from 25.18× to 76.26× across the 20 samples, with an overall mean of 31.67×. The mean coverage was 30.88× for the female samples and 32.46× for the male samples. SsF1 and SsM1, which were sequenced at greater depth for de novo assembly, achieved coverage values of 63.14× and 76.26×, respectively, whereas the remaining 18 samples had a mean coverage of 27.44×. These results indicated that the resequencing data were of high quality and suitable for the subsequent comparative analysis. Comparison of female and male resequencing data identified 55 candidate male-associated fragments, ranging from 102 to 2759 bp in length (Figure 1). At this stage, these fragments had not yet been anchored to the reference genome; therefore, their chromosomal locations and genomic distribution were not determined.

### 3.2. Identification and Evaluation of Candidate Male-Associated Markers

Primers were designed for the candidate fragments and initially screened using genomic DNA from five males and five females, yielding 21 candidates with male-specific amplification (Figure 2). In an independent expanded set of 20 males and 20 females, seven markers (Marker4, Marker5, Marker18, Marker22, Marker23, Marker29, and Marker32) were amplified in all 20 males and in none of the 20 females (Figure 3). Within this cohort, each marker therefore had an apparent amplification sensitivity of 100%, an apparent amplification specificity of 100%, and an overall concordance of 100% (40/40). The sex-by-amplification association showed complete separation (uncorrected odds ratio = ∞; two-sided Fisher exact *p* = 1.45 × 10^−11^). These descriptive values apply only to the tested single-farm cohort and should not be interpreted as estimates of population-wide diagnostic performance.

**Figure 2:**
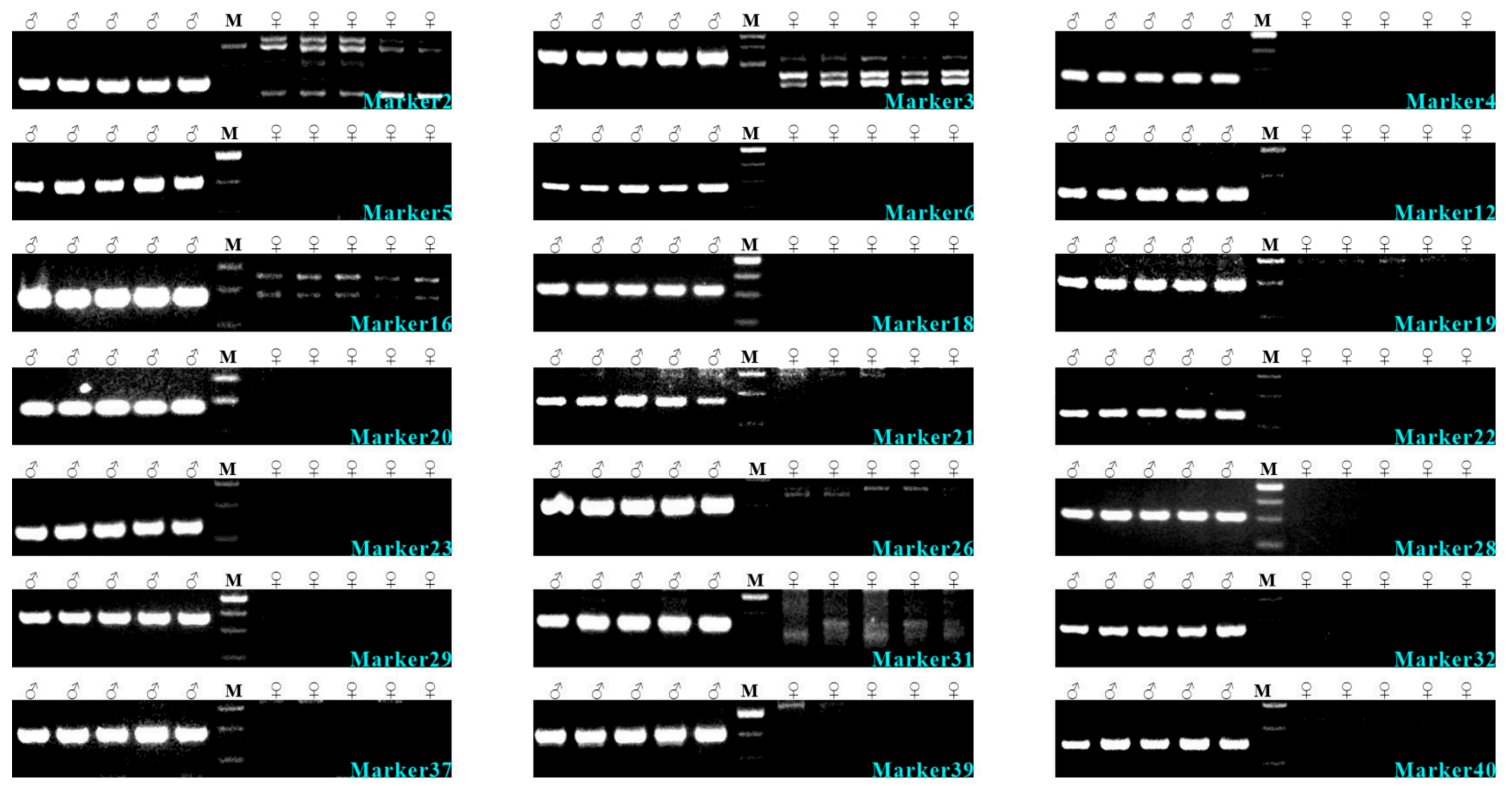
Initial PCR screening of candidate male-associated markers in *S. scherzeri*. M denotes the DL1000 DNA molecular-weight marker. The original assays did not include a dedicated no-template control or a co-amplified internal amplification control.

**Figure 3:**
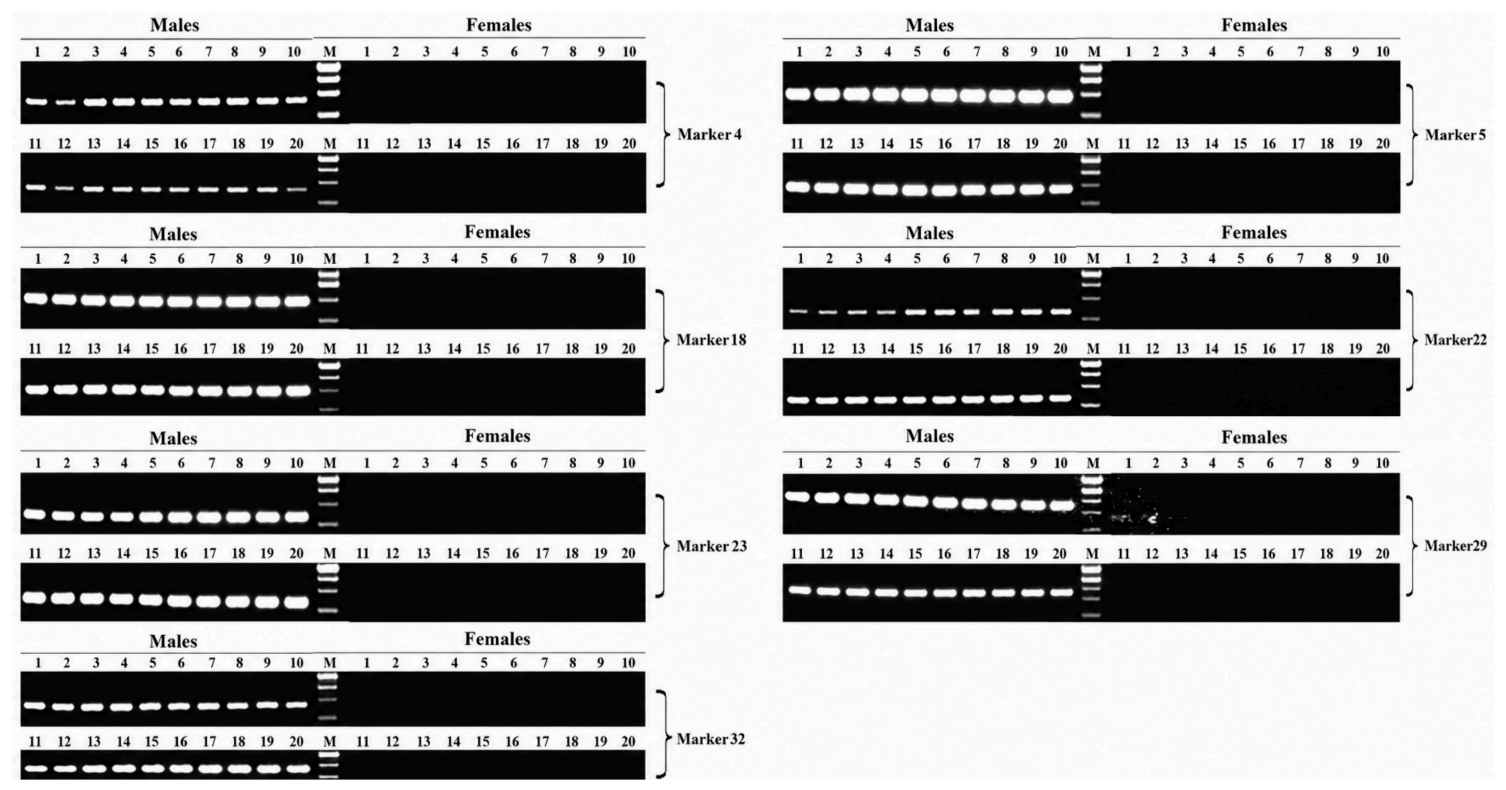
PCR evaluation of seven candidate male-associated markers in an independent set of 20 males and 20 females of *S. scherzeri*. M denotes the DL1000 DNA molecular-weight marker; predicted amplicon lengths are listed in Table 2. The original assays did not include a dedicated no-template control or a co-amplified internal amplification control.

**Table 2:** Genomic coordinates and within-cohort amplification performance of seven candidate male-associated markers.

Marker	Chr14 Coordinate (bp)	Amplicon (bp)	Male + (*n*/*N*)	Female − (*n*/*N*)	Concordance	Fisher *p*
Marker4	20,677,477–20,677,664	188	20/20	20/20	100% (40/40)	1.45 × 10^−11^
Marker5	20,677,726–20,678,004	279	20/20	20/20	100% (40/40)	1.45 × 10^−11^
Marker22	20,686,331–20,686,563	233	20/20	20/20	100% (40/40)	1.45 × 10^−11^
Marker18	20,688,591–20,688,886	296	20/20	20/20	100% (40/40)	1.45 × 10^−11^
Marker32	20,691,588–20,691,859	272	20/20	20/20	100% (40/40)	1.45 × 10^−11^
Marker29	20,692,685–20,693,053	369	20/20	20/20	100% (40/40)	1.45 × 10^−11^
Marker23	20,695,193–20,695,425	233	20/20	20/20	100% (40/40)	1.45 × 10^−11^

Note: Coordinates refer to CM148001.1 (GCA_055815275.1). All markers showed complete separation in the expanded cohort. The uncorrected odds ratio was infinite because both discordant cells were zero; the two-sided Fisher exact *p* value is reported. These values are descriptive of the tested cohort and are not estimates of population-level diagnostic performance.

### 3.3. Preliminary Cross-Species Evaluation of Candidate Male-Associated Markers

To preliminarily evaluate the cross-species amplification patterns of the seven candidate male-associated markers, PCR assays were performed in the tested samples of two additional Siniperca species, *S. chuatsi* and *S. knerii*. Marker4, Marker5, and Marker18 were amplified in all eight males and none of the eight females examined for each species (Figure 4). These results indicate male-associated amplification in the tested samples only. The small sample size and single-source sampling design do not establish genus-wide conservation or diagnostic performance, and broader population-level validation with appropriate PCR controls is required.

### 3.4. Genomic Localization of a Marker-Enriched Male-Associated Interval

Alignment of the seven PCR-tested amplicons to the *S. scherzeri* reference assembly identified a single predicted reference amplicon for each primer pair on chromosome 14 (CM148001.1). Their homologous reference positions spanned 20,677,477–20,695,425 bp, defining a 17,949-bp marker-enriched interval (Figure 5; Table 2). Marker18 was predicted to span 20,688,591–20,688,886 bp and overlapped 293 bp of the partial *amh*-family model ACWW37_011111 (20,688,594–20,689,210 bp, minus strand), predominantly within its second annotated exon/CDS. The reference GFF describes ACWW37_011111 as a partial hypothetical protein, and the reference assembly was generated from an XX female. Consequently, sequence similarity and positional overlap are insufficient to identify this model as a male-specific *amhy* duplicate. The present data do not demonstrate a male-specific nucleotide variant, copy-number difference, structural variant, expression pattern, or functional role associated with this model. They also do not establish that the marker-enriched interval is Y-specific, recombination-suppressed, or itself an SDR.

## 4. Discussion

### 4.1. Marker Development, Within-Cohort Evaluation, and Preliminary Cross-Species Applicability

Because chromosome 14 and sex-associated markers had already been reported in *S. scherzeri*, the principal contribution of the present study is not the initial identification of a sex-associated chromosome. Li et al. developed one male-specific and one female-specific marker following GWAS and *F_ST_* analyses, whereas the present read-presence/absence strategy yielded seven additional candidate male-associated markers. All seven showed complete separation in the present expanded cohort, but no direct comparison with previously published markers was performed in the same individuals. We therefore cannot conclude that the new markers provide higher accuracy or reliability. Their potential value lies in providing several complementary loci for future assay development, but this potential advantage remains to be evaluated in independent families and populations with appropriate negative and internal amplification controls. Three markers also showed male-associated amplification in the tested *S. scherzeri*, *S. chuatsi*, and *S. knerii* samples. Earlier work had already reported male-associated genomic regions and markers across mandarin fishes [31], and more recent research implicated *amhy* in the sex-determination system of Sinipercidae [34]. Thus, the present results are preliminary independent observations rather than evidence of genus-wide conservation. Larger samples from multiple populations of each species and direct comparisons with existing markers will be required to determine reproducibility and potential practical value.

### 4.2. Genomic Localization and Conservative Interpretation of the amh-Family Overlap

Li et al. [30] combined genome-wide association and *F_ST_* analyses and proposed chromosome 14 as the sex-linked chromosome of *S. scherzeri*, identifying a 0.44-Mb sex-linked region at 19.34–19.78 Mb. All seven markers identified in the present study had homologous reference positions on chromosome 14, but they clustered within a non-overlapping 17,949-bp interval at 20.677–20.695 Mb, approximately 0.90 Mb downstream of the previously reported region. This chromosome-level agreement was noteworthy, but our data did not independently establish chromosome 14 as a differentiated sex chromosome. The biological relationship between these two non-overlapping signals cannot be resolved using the present data. Genome-wide association and *F_ST_* analyses detect sex-associated allele-frequency differentiation [35,36], whereas our read-presence/absence approach was designed to identify short sequences consistently detected in males but not females. Therefore, the two approaches detect different types of genomic signals, and the present marker-discovery strategy cannot define SDR boundaries or evaluate linkage disequilibrium and recombination suppression. From a biological perspective, population- or family-specific male-associated haplotypes, recombination within a young sex-associated region, or structural variation could potentially contribute to the positional discrepancy [37,38,39,40]. However, none of these possibilities was directly examined in the present study, and they should therefore be regarded as hypotheses rather than demonstrated explanations. Furthermore, because GCA_055815275.1 was assembled from an XX female, these coordinates represent homologous positions in the female reference rather than a resolved male haplotype. Accordingly, the present data cannot determine whether the marker cluster represents an additional male-linked region, an extension of the previously proposed SDR, or a positional discrepancy resulting from the reference assembly and short-read mapping. Therefore, the interval identified here should be described as a male-associated marker-enriched reference interval, rather than an extension of, a second locus adjacent to, or a redefinition of the previously reported SDR.

Members of the *amh* gene family, including independently evolved male-specific *amhy* duplicates, have repeatedly been implicated in male sex determination and testicular differentiation in teleost fishes [19,41]. Previous work in Sinipercidae has also proposed an *amhy*-associated mechanism [34]. In the present study, Marker18 overlapped 293 bp of the partial gene model ACWW37_011111. However, positional overlap alone does not link the marker to a causal sex-determining gene. The reference GFF describes ACWW37_011111 as a partial hypothetical protein, and the reference genome was assembled from an XX female [32]. The present study did not determine a complete gene sequence, demonstrate a male-specific nucleotide or structural variant, assess sex-specific copy number, examine expression during gonadal differentiation, or test biological function. Accordingly, the *amh*-family overlap is treated only as a genomic annotation requiring further genetic and functional evaluation. We do not infer sex-determination roles for other genes near the interval because physical proximity, without sex-associated variants, sex-biased expression, or functional evidence, is insufficient. The present results identify a male-associated marker cluster on chromosome 14 but do not establish a differentiated sex chromosome, define an SDR, or identify a master sex-determining gene.

### 4.3. Potential Applications

Beyond their value as research markers, the seven candidate markers could potentially support early genetic sex identification in *S. scherzeri* if their performance is independently confirmed. Because the assays use genomic DNA, future testing could use nonlethal fin-clip samples before phenotypic sex can be reliably determined. However, the complete separation observed in 20 males and 20 females is an initial within-cohort observation rather than established diagnostic accuracy. Moreover, the original PCR assays lacked dedicated no-template controls and co-amplified internal amplification controls. Thus, contamination was not monitored by a concurrent no-template reaction, and the absence of a marker band could not be distinguished unequivocally from PCR failure or poor DNA quality within the same sample. This limitation is particularly important for a presence/absence assay and prevents strong claims of diagnostic reliability. Future validation should include no-template controls, known positive controls, a co-amplified endogenous internal control, and preferably more than one independently validated male-associated marker. Until reproducibility has been demonstrated across independent families, farms, and geographic populations, these markers should be considered research-stage male-associated markers rather than validated diagnostic tools for routine commercial application.

### 4.4. Limitations and Future Directions

Several limitations constrain the interpretation and generalization of this study. First, the whole-genome discovery dataset included only 10 males and 10 females, while expanded PCR evaluation included 20 males and 20 females. All *S. scherzeri* individuals originated from a single farmed cohort, and pedigree information was unavailable. Undetected relatedness or a family- or population-specific male haplotype may therefore have contributed to the complete marker–sex association. The cross-species sets were also small and originated from a single source. Consequently, the observed amplification patterns should not be extrapolated to entire species or geographic populations. Second, the original PCR assays included neither dedicated no-template controls nor co-amplified internal amplification controls. A negative marker band therefore could not be distinguished unequivocally from amplification failure, and concurrent contamination monitoring was unavailable. This limits interpretation of the presence/absence results and precludes claims of established diagnostic reliability. Third, the short-read presence/absence strategy was designed for marker discovery rather than comprehensive characterization of sex-associated genomic architecture. It did not evaluate genome-wide sex-associated SNPs, copy-number variation, large structural variants, linkage disequilibrium, or recombination suppression. Mapping male-derived short sequences to an XX female reference also cannot determine the structure or physical location of the corresponding male haplotype. Thus, the present data neither delimit an SDR nor demonstrate chromosome-wide X–Y differentiation. Fourth, the relationship between Marker18 and the partial *amh*-family model is based solely on homologous reference-coordinate overlap. The amplicons were not used to establish a complete male-specific gene sequence, and no copy-number, transcriptional, developmental-expression, or functional analysis was performed. Therefore, the identity of the master sex-determining gene remains unresolved. Future studies should include independent populations and informative families, PCR assays with appropriate no-template and internal controls, haplotype-resolved long-read sequencing of males, genome-wide SNP and structural-variant analyses, linkage mapping, gonadal expression profiling, and functional validation.

## 5. Conclusions

In summary, whole-genome resequencing and PCR testing identified seven candidate male-associated markers that showed male-specific amplification and complete concordance with phenotypic sex in 20 males and 20 females from a single farmed cohort. Their homologous reference positions clustered within a 17,949-bp interval on chromosome 14, and Marker18 overlapped a partial *amh*-family gene model. These findings nominate a male-associated marker-enriched interval for further investigation. However, the single-cohort design, unavailable pedigree information, and absence of dedicated no-template and internal amplification controls mean that the markers cannot yet be regarded as broadly validated diagnostic tools. The data also do not establish chromosome 14 as a differentiated sex chromosome, define the interval as an SDR, or demonstrate that the overlapping *amh*-family model is a master sex-determining gene. Independent population validation with appropriate PCR controls, together with structural, linkage, expression, and functional analyses, will be required.

## Figures and Tables

**Figure 1:**
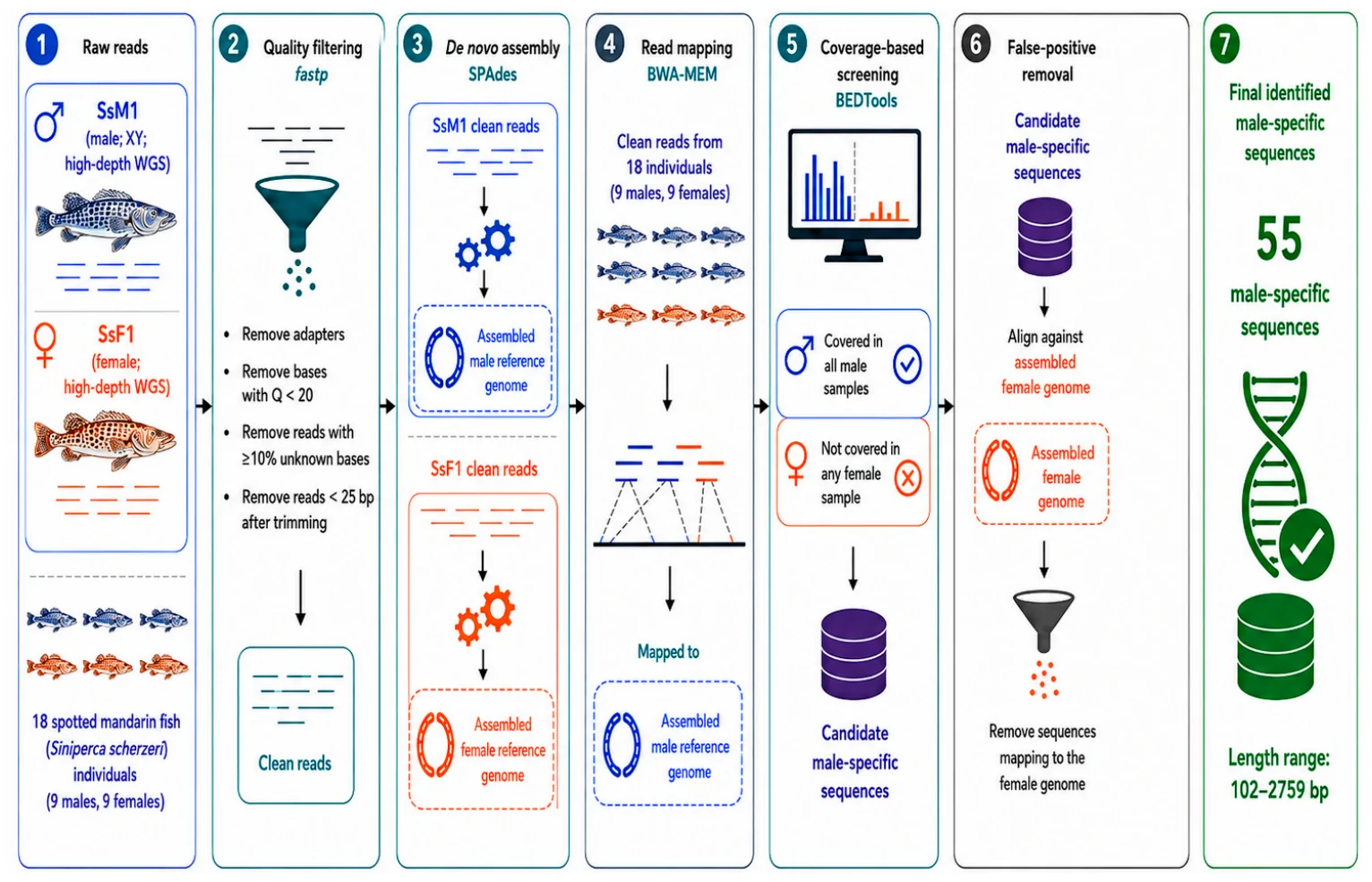
Workflow for screening candidate male-associated fragments in *S. scherzeri*. Black arrows indicate the successive steps of the analytical workflow. Blue and orange elements represent male- and female-derived reads or genome assemblies, respectively, while green indicates the final retained male-specific sequences. The symbols ♂ and ♀ denote male and female samples, respectively. Dashed lines indicate read-mapping relationships. A check mark indicates coverage in all male samples, whereas a cross indicates the absence of coverage in all female samples.

**Figure 4:**
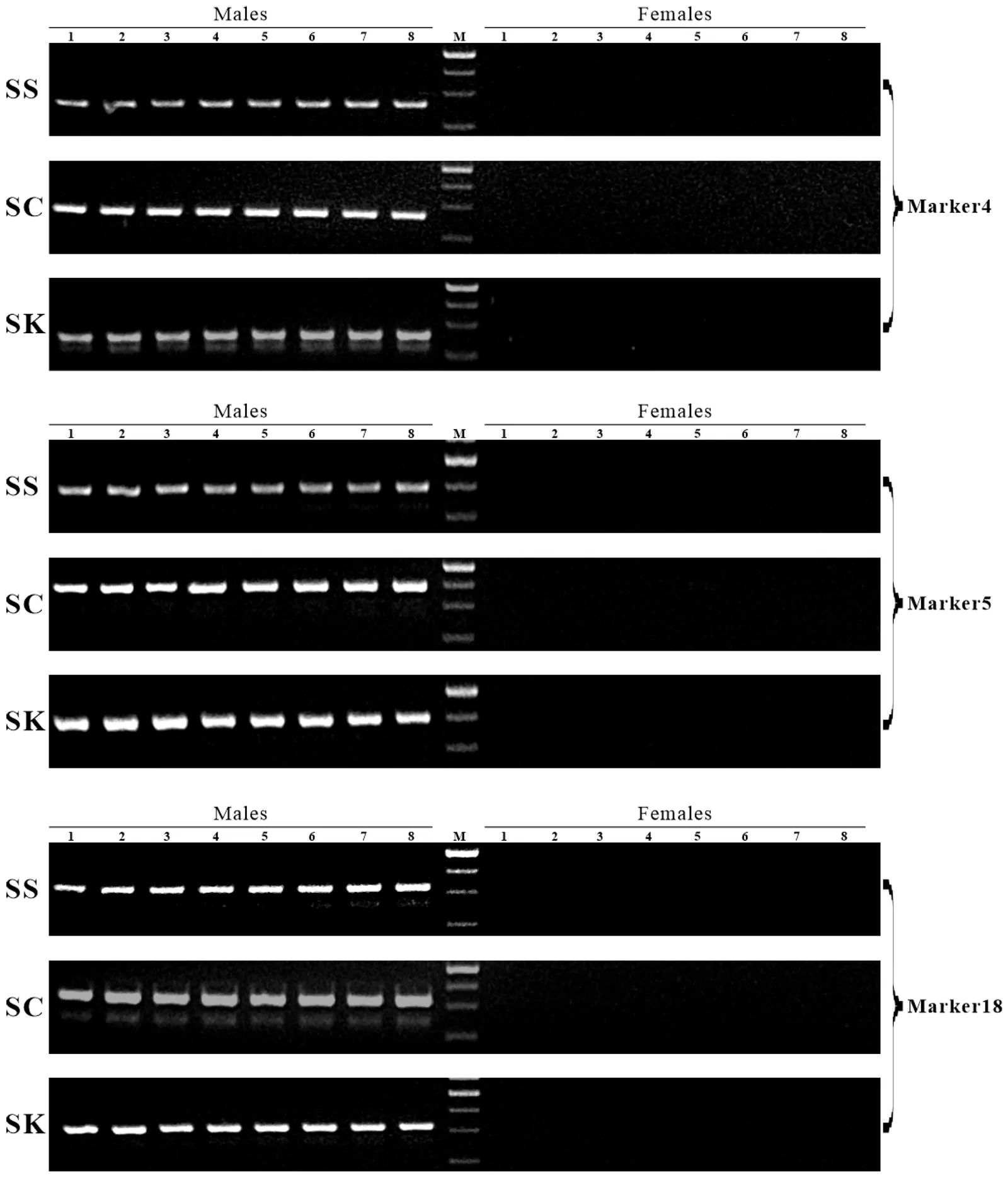
Preliminary cross-species PCR evaluation of three candidate male-associated markers in the tested Siniperca fishes. SS, *S. scherzeri*; SC, *S. chuatsi*; SK, *S. knerii*. Male and female lanes are numbered 1–8; M denotes the DL1000 DNA molecular-weight marker. The original assays did not include a dedicated no-template control or a co-amplified internal amplification control.

**Figure 5:**
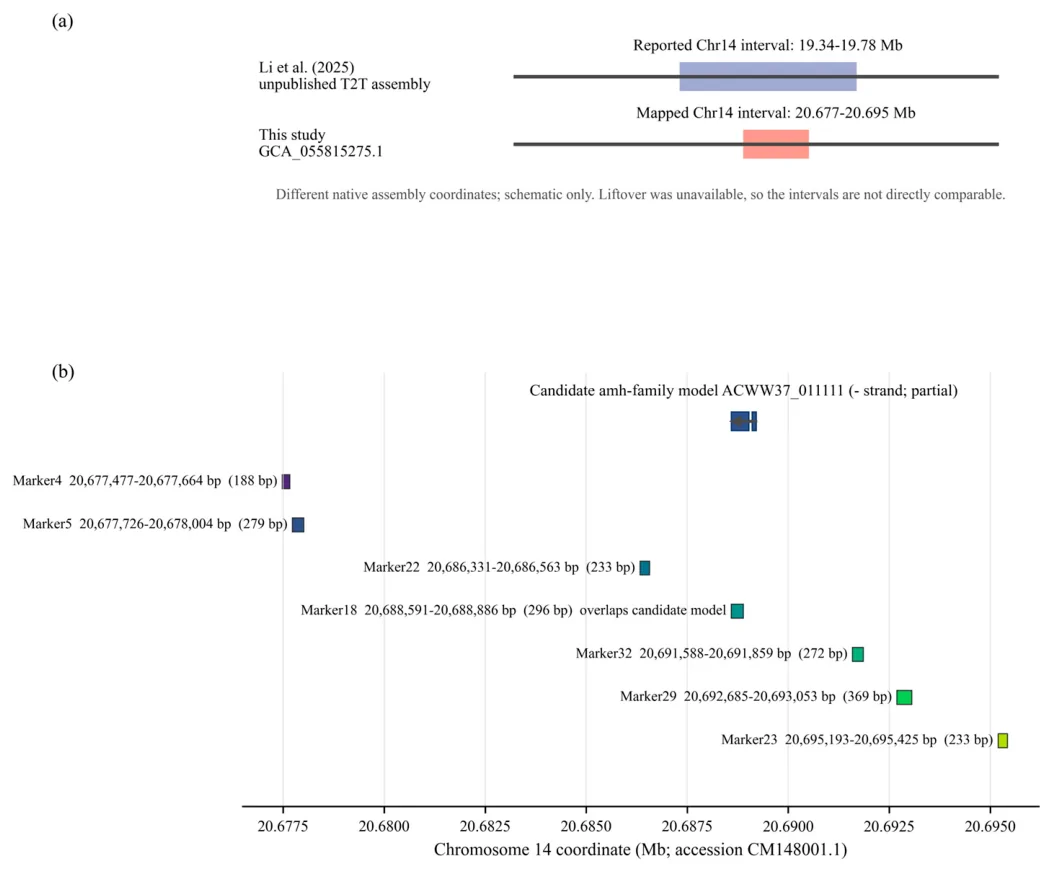
Genomic positions of seven candidate male-associated markers on chromosome 14 of *S. scherzeri*. (**a**) Comparison between the previously reported putative sex-determining region (19.34–19.78 Mb) [30] and the marker-enriched interval identified in this study (20.677–20.695 Mb). (**b**) Exact reference-genome positions and predicted amplicon lengths of the seven markers. Marker18 spans 20,688,591–20,688,886 bp and overlaps the partial candidate *amh*-family model ACWW37_011111 (20,688,594–20,689,210 bp; minus strand). The reference accession is CM148001.1 (GCA_055815275.1). The partial gene model is designated here as an *amh*-family model; its sequence identity, sex specificity, expression, and function require further validation.

**Table 1:** Summary statistics of whole-genome resequencing.

Sample	Raw Reads	Raw Bases	Clean Reads	Clean Bases	Q20	Q30	GC	Coverage (×)
F1	160,136,336	48,361,173,472	158,855,410	47,850,509,198	98.77%	94.26%	41.36%	63.14
F2	70,950,644	21,427,094,488	70,379,932	21,191,401,853	98.80%	94.31%	40.81%	27.96
F3	69,712,900	21,053,295,800	69,145,889	20,826,479,478	98.73%	94.07%	41.34%	27.48
F4	67,557,949	20,402,500,598	67,046,839	20,201,423,877	98.82%	94.44%	41.02%	26.65
F5	70,623,678	21,328,350,756	70,090,332	21,116,557,377	98.80%	94.34%	40.98%	27.86
F6	63,869,279	19,288,522,258	63,367,601	19,084,774,913	98.71%	93.96%	41.43%	25.18
F7	68,796,164	20,776,441,528	68,290,921	20,573,681,047	98.86%	94.60%	40.93%	27.15
F8	67,376,275	20,347,635,050	66,884,766	20,149,972,511	98.79%	94.28%	41.31%	26.59
F9	70,724,436	21,358,779,672	70,146,310	21,131,613,601	98.92%	94.90%	40.81%	27.88
F10	73,122,048	22,082,858,496	72,609,395	21,878,935,321	98.92%	94.86%	41.01%	28.87
M1	193,268,249	58,367,011,198	191,819,275	57,801,685,719	98.99%	95.14%	41.03%	76.26
M2	66,071,260	19,953,520,520	65,553,751	19,750,511,894	98.86%	94.61%	40.84%	26.06
M3	73,098,062	22,075,614,724	72,480,787	21,833,128,541	98.79%	94.35%	40.92%	28.81
M4	73,889,170	22,314,529,340	73,338,494	22,089,961,330	98.71%	93.95%	41.45%	29.15
M5	66,549,619	20,097,984,938	65,966,940	19,864,969,647	98.75%	94.20%	41.49%	26.21
M6	68,645,246	20,730,864,292	68,088,924	20,508,424,372	98.81%	94.45%	41.35%	27.06
M7	66,092,876	19,960,048,552	65,593,794	19,761,728,686	98.74%	94.04%	41.28%	26.07
M8	73,333,840	22,146,819,680	72,786,434	21,928,602,403	98.83%	94.46%	41.23%	28.93
M9	68,187,847	20,592,729,794	67,680,662	20,390,595,488	98.78%	94.26%	41.11%	26.90
M10	73,775,470	22,280,191,940	73,252,308	22,070,837,399	98.82%	94.38%	40.87%	29.12

## Data Availability

The raw whole-genome resequencing data generated in this study have been deposited in the NCBI Sequence Read Archive (SRA) under BioProject accession number PRJNA1495407. The dataset comprises raw sequencing data from 10 male and 10 female *Siniperca scherzeri* individuals. Upon public release, the data will be permanently accessible under BioProject accession PRJNA1495407.

## Supplementary Materials

The following supporting information can be downloaded at: https://www.mdpi.com/article/10.3390/biology15191675/s1, Table S1: Allocation of fish samples for different molecular analyses; Table S2: Primer design for PCR screening of candidate sex-specific markers.
