# Supplementary material for: Whole-Genome Re-Sequencing Identifies Male-Associated Markers and a Marker-Enriched Region on Chromosome 14 in Siniperca scherzeri
Source: Biology (Basel). 2026 Sep 22;15(19):1675. doi: 10.3390/biology15191675 (PMC13663359; doi:10.3390/biology15191675)
Supplement: Supplementary file 1 [file biology-15-01675-s001.zip › Table S1-R.pdf]

Species	Analysis	Female (n)	Male (n)	Total (n)
S. scherzeri	Whole-genome resequencing	10	10	20
S. scherzeri	First-round PCR validation	5	5	10
S. scherzeri	Expanded PCR validation	20	20	40
S. scherzeri	Cross-species marker validation	8	8	16
S. scherzeri	Backup samples	2	2	4
**Total** *S. scherzeri*		**45**	**45**	**90**
S. chuatsi	Cross-species marker validation	8	8	16
S. knerii	Cross-species marker validation	8	8	16

Table S1. Allocation of fish samples for different molecular analyses
