# Supplementary material for: Whole-Genome Re-Sequencing Identifies Male-Associated Markers and a Marker-Enriched Region on Chromosome 14 in Siniperca scherzeri
Source: Biology (Basel). 2026 Sep 22;15(19):1675. doi: 10.3390/biology15191675 (PMC13663359; doi:10.3390/biology15191675)
Supplement: Supplementary file 1 [file biology-15-01675-s001.zip › Table S2-R.pdf]

Primer Name	Primer sequence (5'-3')	Primer Name	Primer sequence (5'-3')
Marker1-F	GGAATTAagtgcctctgtag	Marker22-F	TATCATACTCTTTATAGTGTT
Marker1-R	gtcaCCTGCGATATCAGCAtg	Marker22-R	GAAAACGCCTTCAACAGTAACC
Marker2-F	TGCTGATATCGCAGGtgactct	Marker23-F	GACCAGCGAGGGCGCTAAACG
Marker2-R	CAAGCACGTGCgtggcatgg	Marker23-R	cccTGTTAAGAGGagatgtt
Marker3-F	cacGCACGTGCTTGaattacac	Marker24-F	tcTTATATACAATGACaca
Marker3-R	agcctttgcACTCcaacat	Marker24-R	TCGCATAGCCAGACTCATCTCC
Marker4-F	CAACAACAAGgatggcga	Marker25-F	tcATTTGTCGTCTGATTaa
Marker4-R	AGAACCCACAATTTCATCTT	Marker25-R	GATCTGTGGGACGATTTGGACC
Marker5-F	ATGGCACTGAAGAAGTTCTTa	Marker26-F	GAATTCAGGGCATGTATTTA
Marker5-R	GTCATGGCACAGGATATTT	Marker26-R	TGGGAAAACCTGCTTCAAAT
Marker6-F	ACTGTATCAGACTTCAAAGC	Marker27-F	GCCTTCAACAGTAACCTGCA
Marker6-R	CACACAGCGCTTAAGTCTGC	Marker27-R	GGGAAACACACATCGGACCCACT
Marker7-F	TGGATGCGTGTGCCAGGAT	Marker28-F	CGGTACCAGAACCTATATCTCTG
Marker7-R	gctGCTCTTAGGTAGCATTtcc	Marker28-R	GAGCAAGTTTACCCTTGGC
Marker8-F	GTGCAAAGCATGCAGTGCA	Marker29-F	TAAGTTCACTGTATGCTAC
Marker8-R	TATCAGACTGCACCCCTTGA	Marker29-R	TTAGCGCTCATTCACATGTTGG
Marker9-F	AAAAGAAGTCCCAAGtccc	Marker30-F	ATTAATTATTAGCACCTAAAT
Marker9-R	acaacccacTCAGCgaac	Marker30-R	CTGGGTCCAGGCTGGGCTTC
Marker10-F	CCTTGATTAGACTGTGTAAGAC	Marker31-F	AGACAAGAACTGATGGAGGT
Marker10-R	tgaagactttggctgaccca	Marker31-R	CCTACAGTGTGTCGGGGAAGC
Marker11-F	CCCCTATGTATTGTCGGTAACCTG	Marker32-F	CCCTCCCGCTCCAGGCTTTGGC
Marker11-R	ttggccctgtgcaccgtggc	Marker32-R	GAATTAAGTGCCTCTGTAG
Marker12-F	TAGcatctctgtgtcatgctg	Marker33-F	ATGAGCGCCACGGATCCGTTCC
Marker12-R	acaCATGGCCGCCAGCTACAT	Marker33-R	GCTCGCCCCTGGAGCTGAG
Marker13-F	ATGTAGCTGGCGGCCATGtgtt	Marker34-F	CCCTGTACCTGACCGAGCAC

Marker13-R	agcatgacacagagatgCTA	Marker34-R	CGCTAGCTGTGGCGATGGT
Marker14-F	TGCAGGATCTGCCTTGA	Marker35-F	Tgtacaaaggtgagaggt
Marker14-R	CGCAGACACCAGTTACGTCAAT	Marker35-R	ctgaatagattCGTAAGCgctc
Marker15-F	TCCTCGGCCATCCCCCTG	Marker36-F	AATTTTATCATTAAGGAAGC
Marker15-R	GACCTGTCATTGTAGTGCT	Marker36-R	ACTACACCTGCCAGTGCAct
Marker16-F	CAAAGGAGACCACCACACAA	Marker37-F	TAAGTGTGTCAATAATTTTACTT
Marker16-R	CATCGCTCCCCACATGTTC	Marker37-R	GTGAGCTGATCCATACTGGG
Marker17-F	GTCTGTGTGCATTTCAGGA	Marker38-F	AATTTTACTTTGAATTGGGCA
Marker17-R	CACCACTCTGATAATAGTCG	Marker38-R	Ttcagtgctgtctgactctgt
Marker18-F	GCCTCCCCTACCACTAGGCTTA	Marker39-F	CCACAGCCAGAAATGAACTTC
Marker18-R	CATCACCTGCTGCTGGATCC	Marker39-R	CATGTAAATTTCCCCCACATGCC
Marker19-F	TCCCACAGCCCTGTCACATGC	Marker40-F	atgactacaactgaaaccttttct
Marker19-R	GTCTTCAACAACACTTAGT	Marker40-R	ACAGAATGTaagacacaa
Marker20-F	agaaTATAACCTacataga	Marker41-F	agttattaaaaccaacaac
Marker20-R	gggccaCCGTCTGcagcgc	Marker41-R	Gacaggatcacagcatcttg
Marker21-F	CAGCGTGTTCAGCATCCACAA	Marker42-F	CCCTATCCCAACTACGCTACT
Marker21-R	CTTATGTGTTAACACTATAAA	Marker42-R	AGAGACAAGTCCATTGTCTGAT

Table S2. Primer design for PCR screening of candidate sex-specific markers.
